# Prostate cancer tumour control probability modelling for external beam radiotherapy based on multi-parametric MRI-GTV definition

**DOI:** 10.1186/s13014-020-01683-4

**Published:** 2020-10-20

**Authors:** Ilias Sachpazidis, Panayiotis Mavroidis, Constantinos Zamboglou, Christina Marie Klein, Anca-Ligia Grosu, Dimos Baltas

**Affiliations:** 1 Department of Radiation Oncology, Division of Medical Physics, Medical Centre, Faculty of Medicine, University of Freiburg, Robert-Koch-Str. 3, 79106 Freiburg, Germany; 2 Department of Radiation Oncology, Medical Centre, University of Freiburg, Freiburg, Germany; 3 German Cancer Consortium (DKTK) Partner Site Freiburg, German Cancer Research Centre (DKFZ), Heidelberg, Germany; 4 Department of Radiation Oncology, University of North Carolina, Chapel Hill, NC USA

**Keywords:** Tumour control probability (TCP), Linear-quadratic Poisson model, Multivariate logistic regression model, Therapy response prediction, Prostate cancer

## Abstract

**Purpose:**

To evaluate the applicability and estimate the radiobiological parameters of linear-quadratic Poisson tumour control probability (TCP) model for primary prostate cancer patients for two relevant target structures (prostate gland and GTV). The TCP describes the dose–response of prostate after definitive radiotherapy (RT). Also, to analyse and identify possible significant correlations between clinical and treatment factors such as planned dose to prostate gland, dose to GTV, volume of prostate and mpMRI-GTV based on multivariate logistic regression model.

**Methods:**

The study included 129 intermediate and high-risk prostate cancer patients (cN0 and cM0), who were treated with image-guided intensity modulated radiotherapy (IMRT) ± androgen deprivation therapy with a median follow-up period of 81.4 months (range 42.0–149.0) months. Tumour control was defined as biochemical relapse free survival according to the Phoenix definition (BRFS). MpMRI-GTV was delineated retrospectively based on a pre-treatment multi-parametric MR imaging (mpMRI), which was co-registered to the planning CT. The clinical treatment planning procedure was based on prostate gland, delineated on CT imaging modality. Furthermore, we also fitted the clinical data to TCP model for the two considered targets for the 5-year follow-up after radiation treatment, where our cohort was composed of a total number of 108 patients, of which 19 were biochemical relapse (BR) patients.

**Results:**

For the median follow-up period of 81.4 months (range 42.0–149.0) months, our results indicated an appropriate *α*/*β* = 1.3 Gy for prostate gland and *α*/*β* = 2.9 Gy for mpMRI-GTV. Only for prostate gland, EQD2 and gEUD2Gy were significantly lower in the biochemical relapse (BR) group compared to the biochemical control (BC) group. Fitting results to the linear-quadratic Poisson TCP model for prostate gland and *α*/*β* = 1.3 Gy were *D*_50_ = 66.8 Gy with 95% CI [64.6 Gy, 69.0 Gy], and *γ* = 3.8 with 95% CI [2.6, 5.2]. For mpMRI-GTV and *α*/*β* = 2.9 Gy, *D*_50_ was 68.1 Gy with 95% CI [66.1 Gy, 70.0 Gy], and *γ* = 4.5 with 95% CI [3.0, 6.1]. Finally, for the 5-year follow-up after the radiation treatment, our results for the prostate gland were: *D*_50_ = 64.6 Gy [61.6 Gy, 67.4 Gy], *γ* = 3.1 [2.0, 4.4], *α*/*β* = 2.2 Gy (95% CI was undefined). For the mpMRI-GTV, the optimizer was unable to deliver any reasonable results for the expected clinical *D*_50_ and *α*/*β*. The results for the mpMRI-GTV were *D*_50_ = 50.1 Gy [44.6 Gy, 56.0 Gy], *γ* = 0.8 [0.5, 1.2], *α*/*β* = 0.0 Gy (95% CI was undefined). For a follow-up time of 5 years and a fixed *α*/*β* = 1.6 Gy, the TCP fitting results for prostate gland were *D*_50_ = 63.9 Gy [60.8 Gy, 67.0 Gy], *γ* = 2.9 [1.9, 4.1], and for mpMRI-GTV *D*_50_ = 56.3 Gy [51.6 Gy, 61.1 Gy], *γ* = 1.3 [0.8, 1.9].

**Conclusion:**

The linear-quadratic Poisson TCP model was better fit when the prostate gland was considered as responsible target than with mpMRI-GTV. This is compatible with the results of the comparison of the dose distributions among BR and BC groups and with the results achieved with the multivariate logistic model regarding *gEUD*_*2Gy*_. Probably limitations of mpMRI in defining the GTV explain these results. Another explanation could be the relatively homogeneous dose prescription and the relatively low number of recurrences. The failure to identify any benefit for considering mpMRI-GTV as the target responsible for the clinical response is confirmed when considering a fixed α/β = 1.6 Gy, a fixed follow-up time for biochemical response at 5 years or Gleason score differentiation.

## Introduction

Advances in radiotherapy treatment and a better understanding of the prostate cancer radiobiology suggest new approaches in dose fractionation to improve prostate cancer control while decreasing radiation induced toxicity. Many studies have compared various radiation delivery regiments e.g. hypofractionated (greater than 2 Gy per fraction) [1] against standard fractionation (1.8 Gy to 2 Gy per fraction) or 3D-CRT versus IMRT [2–6], which indicates that the models and estimation of the model parameters play an important role. In addition, magnetic resonance is a versatile and suitable imaging for radiotherapy enabling visualization of the target structures and organs at risks, as well as tissue characterisation, indicating a new era of imaging-guided radiation therapy [7, 8].

The aim of this study was to investigate the applicability of an established tumour control probability (TCP) model to clinical data of PSA relapse (biochemical relapse) after primary radiation therapy with or without androgen deprivation therapy for prostate cancer. The biochemical recurrence after external radiotherapy was defined according to the Phoenix criteria [9]. The outcome data were analysed considering the 3D-dose distributions in both prostate gland (CT based contours) and GTV as delineated on pre-treatment multi-parametric MRI (mpMRI).

Assuming that the imaging based GTV defines the dominant lesion (DIL) in the prostate, its response to the radiation should define the tumour response. This assumption has been considered in several publications [5, 6, 10, 11] investigating the TCP for focal dose escalation based on GTV. Consequently, and in order to investigate the validity of that assumption in predicting tumour response, the dose distribution in GTV was analysed and considered for TCP modelling in addition to prostate gland.

## Materials and methods

### Patient cohort and treatment

Our investigation is based on a retrospective, single institution analysis of all patients with localized and histologically proven prostate cancer (PCa) treated with external beam radiotherapy (EBRT) with or without androgen deprivation therapy (ADT) from February 2008 to October 2016, with a minimum follow-up of 42 months. Patients were excluded from the analysis in case of cN1 or cM1 disease, EBRT of the pelvic lymph nodes and initial PSA serum values above 50 ng/ml. A multi-parametric MR imaging (mpMRI) or PSMA PET/CT at the maximum of 6 months prior to EBRT was mandatory. ADT over 1 month prior to conduction of MRI scans was also an exclusion criterion. The study was approved by the institutional review board. The patient cohort of our investigation included thus a total of 138 patients. The patients’ follow-up interval was every 3 to 6 months for the first 2 years and every 6 to 12 months thereafter with physical examination, PSA measurements and radiological examination if necessary. The Phoenix definition [9] for PSA relapse was used. Detailed description of CTV and PTV definition, of treatment technique and dose parameters for the total groups of patients are available in Zamboglou et al. [5].

From the cohort of 138 patients, 129 had mpMRI prior to EBRT and have been included in current investigation. The median clinical follow-up for the mpMRI group of 129 patients in the current update was 81.4 months (range 42.0–149.0). The present analysis is based on a mixed follow-up time of 42 to 149 months, including a total of 129 patients with 26 biochemical relapse patients. Moreover, the clinical data with a follow-up period of 5-year post radiation treatment were fitted for complementary analysis.

Regarding EBRT for the 129 patients, in 32% 3D-conformal and in 68% intensity-modulated RT (IMRT) was delivered as image-guided RT (IGRT) using daily 2D/2D imaging and at least one cone-beam CT per week. Intraprostatic fiducial markers were implanted in 94% of the patients prior to EBRT. The aimed and the planned median prescription dose to the PTV were 76.0 Gy and 74.0 Gy (range 66.0–78.0 Gy), respectively. The median number of fractions was 38 (range 28–42), and the median dose per fraction was 2.0 Gy (range 1.7–2.7 Gy). In our cohort of 129 patients with mpMRI prior to EBRT, 26 have been classified as biochemical relapse (BR) after radiotherapy.

The pre-existing mpMRI at the point of treatment planning was included only for staging purposes. Retrospectively and within the framework of the current clinical study, the pre-treatment mpMRI registered to the treatment planning CT and mpMRT-GTV was transferred to the CT. The DVHs for mpMRI-GTV together with those for prostate gland were exported for the current work.

The GTV was contoured by two experienced radiation oncologists in consensus under consideration of the PIRADs v2 criteria [12]. The mpMRI-based GTV was considered as the dominant lesion (DIL) defining the response to the treatment. The mpMRI-GTV volume among all patients was median: 2.3 cc, mean: 3.7 cc, min: 0.3 cc max: 38.0 cc, sd: 4.2 cc. The volume of the prostate gland was median: 49.4 cc, mean: 53.1, min: 21.9 cc, max: 187.6 cc and sd: 22.3 cc. Accordingly, the volume fraction of the mpMRI-GTV to the prostate gland was median: 4.9%, mean: 7.5%, min: 0.5%, max: 44.9%, sd: 7.4%.

The differential dose volume histograms (DDVH) with a bin width of 0.1 Gy for both mpMRI-GTV and prostate gland have been calculated and exported from Eclipse (Varian, TPS v15.6).

In our analysis covariates such as ± ADT, PSA serum levels were not accounted based on the analysis of previous work of our group [5], where it has been shown that the aforementioned covariates have no impact on the biochemical control. In addition, we investigated the impact of the time of mpMRI acquisition prior to the treatment. The Cox-regression analysis is presented in the Additional file 1. The results of Cox-regression indicated no higher risk for the patients who had MRI before 30 days comparing to the ones who had MRI after 30 days (*p* = 0.70). On the contrary, it was shown that the Gleason score has a significant impact (*p* = 0.0065) on the treatment response (Additional file 1: Sect. 1). Finally, 68% of the patients had IMRT plans in VMAT technique while the remaining 32% were treated with 3D-CRT. The treatment technique has shown to have no statistically significant effect on the observed results (*p* = 0.08 Cox-regression analysis, see Additional file 1: section 1).

### The generalized equivalent uniform dose gEUD and gEUD_2Gy_

Given the differential dose volume histogram for a specific dose distribution {*D*} in a volume of interest (VOI) the generalized equivalent uniform dose (*gEUD*) can be computed by the following expression [13, 14]:

1 $$gEUD = gEUD\left( {\left\{ D \right\}} \right) = \left( {\mathop \sum \limits_{i = 1}^{N} { }\left( {\frac{{{\varvec{v}}_{{\varvec{i}}} }}{{\mathop \sum \nolimits_{{{\varvec{k}} = 1}}^{{\varvec{N}}} {\varvec{v}}_{{\varvec{k}}} }}} \right)D_{i}^{\alpha } } \right)^{{{\raise0.7ex\hbox{$1$} \!\mathord{\left/ {\vphantom {1 \alpha }}\right.\kern-\nulldelimiterspace} \!\lower0.7ex\hbox{$\alpha $}}}}$$

*N* is the number of bins of the differential DVH of the corresponding VOI, tumour or organ at risk (OAR), *D*_*i*_ is the dose and *v*_*i*_ is the volume at the *i*th bin. $\sum_{k=1}^{N}{v}_{k}=V$, i.e. the volume of the VOI. Parameter *α* (*α* < 0 for tumours and *α* > 0 for normal tissues) is the specific parameter that describes the dose-volume effect of the anatomic structure of interest.

*gEUD* is based on the physical three-dimensional (3D) dose distribution {*D*} and is the dose which when delivered homogeneously to the volume of interest will result in the same biological effect as the inhomogeneous dose distribution described of the underlying DDVH.

To account for the differences in biological effectiveness of the different dose levels at different sampling points within the VOI, the *gEUD*_*2Gy*_ quantity can be used [14]. *gEUD*_*2Gy*_ uses the 2 Gy per fraction equi-effective dose distribution and is calculated in a similar way to *gEUD* as:

2 $$gEUD_{2Gy} = \left( {\mathop \sum \limits_{i = 1}^{N} \left( {\frac{{{\varvec{v}}_{{\varvec{i}}} }}{{\mathop \sum \nolimits_{{{\varvec{k}} = 1}}^{{\varvec{N}}} {\varvec{v}}_{{\varvec{k}}} }}} \right)EQD2_{i}^{\alpha } } \right)^{{{\raise0.7ex\hbox{$1$} \!\mathord{\left/ {\vphantom {1 \alpha }}\right.\kern-\nulldelimiterspace} \!\lower0.7ex\hbox{$\alpha $}}}}$$

*EQD2*_*i*_ is the equi-effective dose at 2 Gy per fraction of a total dose *D*_*i*_ delivered at *d*_*i*_ dose per fraction for the ith DDVH-bin:

3 $$EQD2_{i} = { }\frac{{D_{i} \left( {1 + { }\frac{{d_{i} }}{a/\beta }} \right)}}{{1 + { }\frac{{2{ }Gy}}{a/\beta }}}$$

*α*/*β* in Gy is the fractionation sensitivity parameter of the specific VOI according to the linear-quadratic (LQ) model [15, 16].

### Tumour control probability models

#### Linear-quadratic Poisson TCP model

Tumour control probability (TCP) models are mathematical formulations to predict the tumour response to radiation therapy on the basis of a dose–response relationship. A widely established formulation for describing this dose–response relationship for tumours is the linear-quadratic Poisson model [17]:

4 $$P\left( D \right) = P\left( {EQD2} \right) = \exp \left( { - e^{{e\,\gamma - \left( {\frac{EQD2}{{D_{50} }}} \right){*}\left( {e{ }\gamma - \ln \ln 2} \right)}} } \right)$$

*P*(*D*) is the tumour control probability, when the tumour is homogenously irradiated at the total dose *D* and *EQD2* is the equi-effective dose at 2 Gy per fraction of the total dose *D* when delivered at a fraction dose *d* (see Eq. 3).

*D*_50_ is the dose in Gy, defined as EQD2, which results in a TCP value of 50% and *γ* is a dimensionless parameter, defining the maximum normalized value of the dose–response gradient.

For an inhomogeneous dose distribution {*D}* within the tumour of volume *V*, the overall tumour control probability *TCP* is calculated according to:

5 $$TCP\left( {\left\{ D \right\},V} \right) = { }\mathop \prod \limits_{i = 1}^{N} \left[ {P\left( {D_{i} } \right)} \right]^{{v_{i} /V}}$$

Or considering Eq. 4 for *P*(*D*_*i*_)

6 $$TCP\left( {\left\{ D \right\},V} \right) = { }\mathop \prod \limits_{i = 1}^{N} \left[ {\exp \left( { - e^{{e\,\gamma - \left( {\frac{{EQD2_{i} }}{{D_{50} }}} \right){*}\left( {e{ }\gamma - \ln \ln 2} \right)}} } \right)} \right]^{{v_{i} /V}}$$

*N* is the total number of tumour sub-volumes *v*_*i*_ each of which is assumed to be irradiated homogeneously at the total dose *D*_*i*_ with an equi-effective dose value *EQD2*_*i*_. Since the dose distribution within the tumour {*D*} is commonly described by the differential DVH, in this case *N* is the total number of dose-bins used for the DDVH calculation.

#### Multivariate logistic regression

In multivariate logistic regression the depending variable (*Y*) is given as a function of several independent (*X*_*i*_) variables in the form of:

7 $$Y = \frac{1}{{1 + e^{{ - (b_{0} + \sum b_{i} X_{i} )}} }}$$

The main null hypothesis of a multivariate logistic regression is that there is no relationship between the *X*_*i*_ variables and the *Y* variable: H_0_: b_i_ = 0, which means that the predicted Y values of the logistic model equation are no closer to the actual Y values than you would expect by chance (if *b*_*i*_ = 0 then *Y* = 1/(1 + 1) = 0.5). Putting it another way, in a multivariate logistic regression we are studying if the independent *X*_*i*_ variables have an effect on the probability of obtaining a particular value of the dependent *Y* variable. TCP can also be described using a multivariate logistic regression model [18, 19] where the independent variables, for example, can be the age, the treatment dose, the volume of the target. Accounting for the inhomogeneity of dose distribution in the target volume we consider the equivalent uniform dose *gEUD*_*2Gy*_ instead of the physical prescription total treatment dose. Considering both target volumes, prostate gland and GTV, as well as the Gleason score, the multivariate logistic regression formulation for TCP (full model) is the following [20]:

8 $${\text{TCP}} = 1 - {\text{Y}} = { }\frac{1}{{1 + e^{{(b_{0} + {\text{ b}}_{1} {\text{gEUD}}_{{2{\text{Gy}},{\text{ prostate}}}} + {\text{ b}}_{2} {\text{ gEUD}}_{{2{\text{Gy}},{\text{GTV}}}} + {\text{ b}}_{3} {\text{ V}}_{{{\text{prostate}}}} + b_{4} V_{GTV} + b_{5} GleasonScore )}} }}$$

### Model fitting

The linear-quadratic Poisson TCP model was fitted using the maximum likelihood estimation (MLE) technique. The likelihood function *L* for the binomial model (response *r* = 1 for relapse-free (BRFS) and 0 for relapse) is:

9 $$L\left( P \right) = L\left( {\left( {D_{50} ,\gamma ,\frac{\alpha }{\beta }} \right), \left( {\left\{ D \right\},V} \right)} \right) = \mathop \prod \limits_{j = 1}^{N} P_{j}^{{r_{j} }} \cdot (1 - P_{j} )^{{\left( {1 - r_{j} } \right)}}$$

with *P*_*j*_ the TCP prediction, *r*_*j*_ the binary clinical response for the *j*th patient and *N* the total number of patients in the study. The best parameter estimation for *D*_50_ and *γ* are those maximizing the *L*(*P*) estimator or equivalently minimizing the *LL* = *−*ln(*L*(*P*)):

10 $$minimize \left\{ {LL} \right\} = minimize\left\{ { - \mathop \sum \limits_{j = 1}^{M} \left\{ {r_{j} \ln \left( {P_{j} } \right) + \left( {1 - r_{j} } \right)\ln \left( {1 - P_{j} } \right)} \right\}} \right\}$$

For the optimization we used simulated annealing (SA), a stochastic solver as implemented in open source “Object Oriented Optimization Toolbox” .NET library [21]. The estimation of the confidence interval (CI) for the parameter values was based on the likelihood profiling method, without assuming normality of the maximum likelihood estimator [22].

For the multivariate logistic regression model, the glm() fitting function and stepwise selection stepAIC() function, in both directions, as provided by R version 3.5.3 [23, 24] were used. Akaike Information Criterion (AIC) was considered to measure the relative quality of the nested models in multivariate analysis [25, 26].

### Goodness-of-fit

For the goodness-of-fit of our models, the Hosmer–Lemeshow (HL) test [27] was performed to test the hypothesis that the predictions agree well with the observed outcomes, in which a *p* value of greater than 0.05 indicates good agreement [28]. A group parameter value of *g* = 10 was used. HL test performed with *ResourceSelection* package [29] in R version: 3.5.3.

### Model parameter values and assumptions

For the TCP model fitting and for the mpMRI-GTV and prostate gland *α*/*β* values in the range of 0.1 Gy to 20.0 Gy have been considered. The optimizer searched for the optimal solution, in a space defined by D_50_, γ and α/β in range of 0.0 Gy to 100.0 Gy, and 0.0 to 10.0, and 0.0 Gy to 20.0 Gy, respectively.

For the generalized equivalent uniform dose *gEUD*_*2Gy*_, a value of *α* = − 10 was applied [14] for both target types; prostate gland and GTV. Plots created in Python 3.7.7 with matplotlib (v3.1.3), numpy (1.18.1), pandas (1.0.3), rpy2 (2.9.4), pyradiobiology (1.0.33), pydvh (1.0.8) libraries. All statistical comparisons were performed with Wilcoxon rank sum nonparametric test (R package stats version 3.6.2) with a significance level (alpha) of 0.05.

## Results

### Comparison of radiobiological dose in prostate gland and mpMRI-GTV

We compared the minimum EQD2 dose value for the two target types, mpMRI-GTV and prostate gland, for the biochemical relapse (BR) and biochemical control (BC) groups. As it will be discussed later and based on the linear-quadratic Poisson TCP model fitting, the most appropriate *α*/*β* value was 1.3 Gy and 2.9 Gy for prostate gland and mpMRI-GTV, respectively.

For the BC group the median of the minimum EQD2 in the prostate gland was 70.6 Gy (range 43.0–82.2 Gy) compared to 72.9 Gy (range 57.5–79.3 Gy) for the mpMRI-GTV, and for the BR group it was 68.7 Gy (range 43.0–77.3 Gy) and 72.1 Gy (range 46.1–76.8 Gy) respectively.

**Fig. 1:**
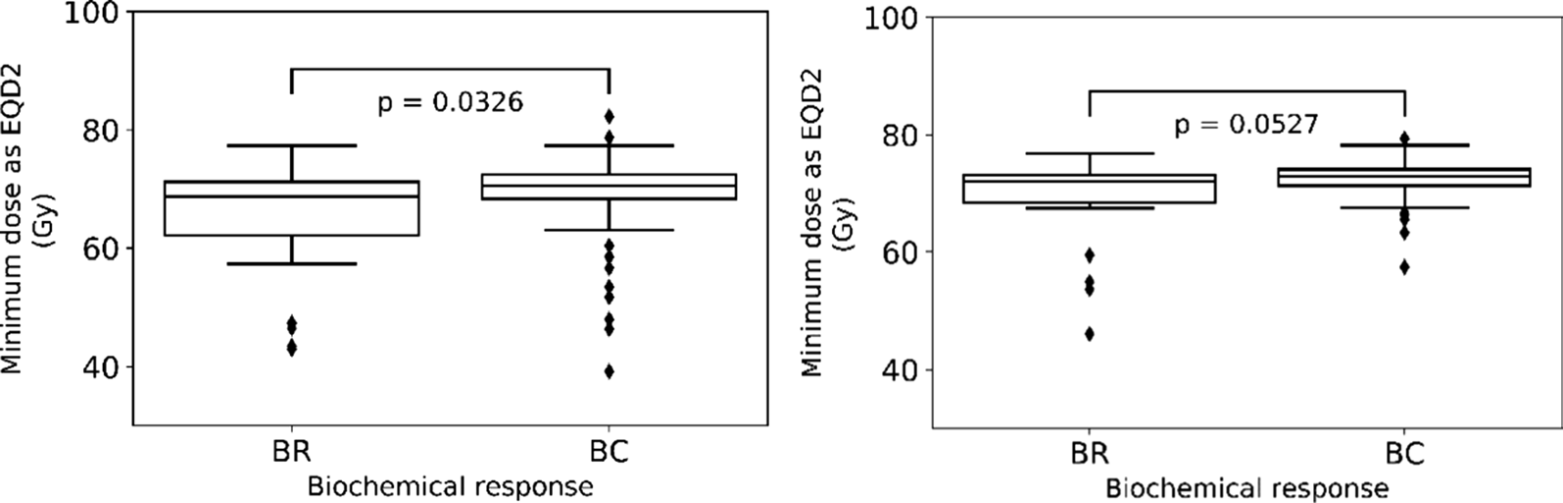
Boxplots of the minimum dose as EQD2 for prostate gland with *α*/*β* = 1.3 Gy (left) and mpMRI-GTV with *α*/*β* = 2.9 Gy (right) for the biochemical relapse (BR) and biochemical control (BC) groups

Figure 1 illustrates the boxplot of the minimum dose as EQD2 in mpMRI-GTV and prostate gland. Similarly, to the analysis for the minimum physical dose (Fig. 1), significantly lower minimum EQD2 values were observed in the BR group compared to the BC group only for prostate gland (*p* = 0.0326).

The median *gEUD*_*2Gy*_ for the two target types, prostate gland and mpMRI-GTV, was 75.3 Gy (range 63.9–86.1 Gy) and 75.3 Gy (range 68.9–81.6 Gy) for the BR group and 74.5 Gy (range 58.5–79.1 Gy) and 74.3 Gy (range 60.3–78.2 Gy) for the BC group, accordingly.

**Fig. 2:**
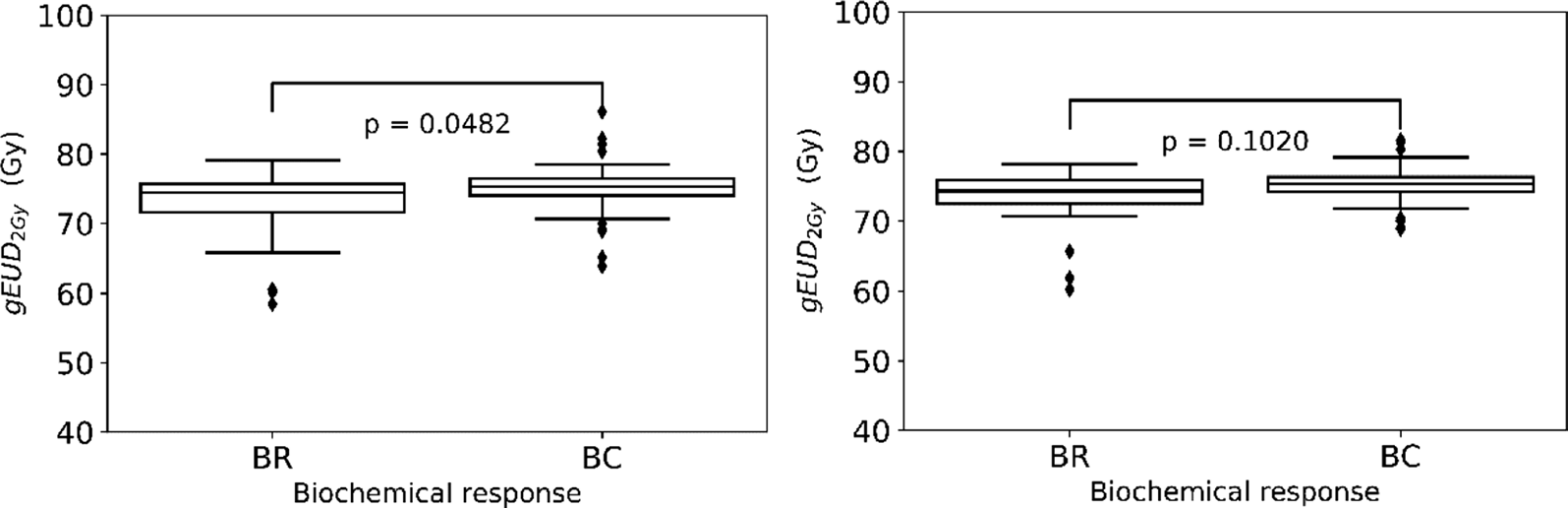
Boxplots of the generalized equivalent uniform dose, *gEUD*_*2Gy*_, for prostate gland (left) with *α*/*β* = 1.3 Gy and mpMRI-GTV (right) with *α*/*β* = 2.9 Gy for the biochemical relapse (BR) and biochemical control (BC) groups

When comparing the *gEUD*_*2Gy*_ values for the two response groups (BR and BC) and the two target types, prostate gland and GTV, only for the prostate gland, a marginally significant lower *gEUD*_*2Gy*_ value in the BR group could be demonstrated (*p* = 0.0482, Fig. 2). This is in alignment with our previous findings for EQD2 and physical dose.

**Fig. 3:**
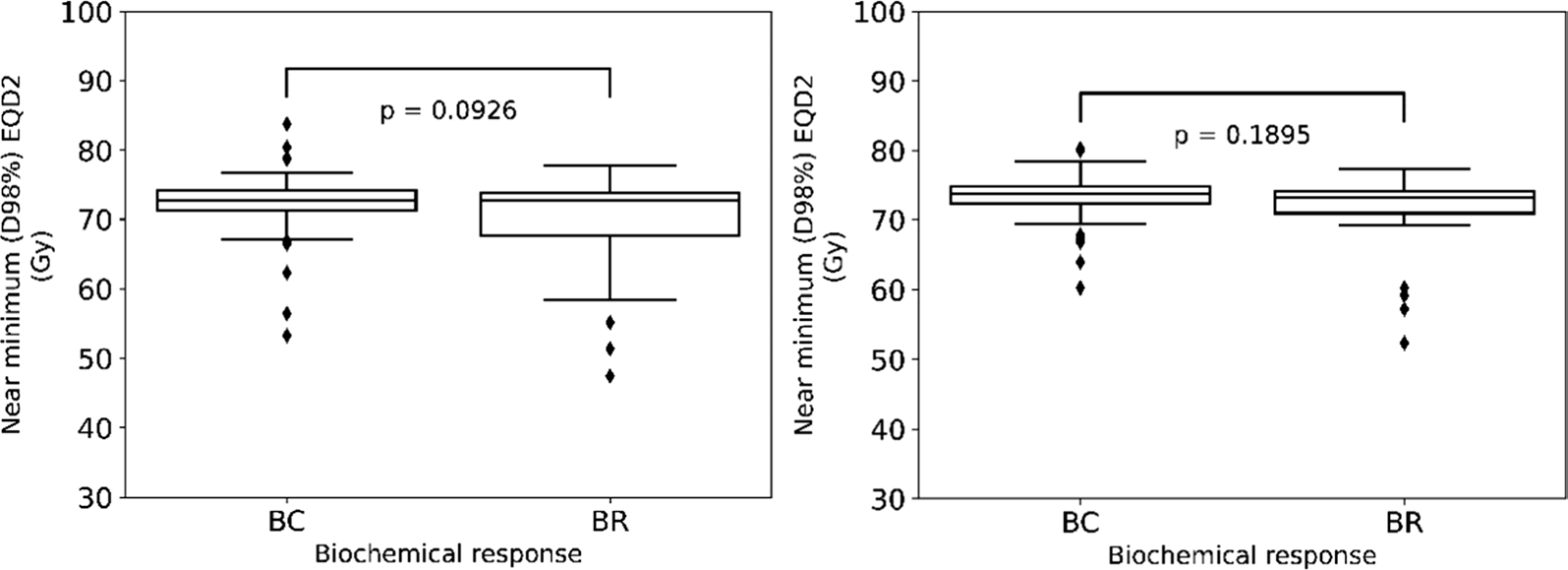
Boxplots for the near minimum (D98%) EQD2 for prostate gland (left) and mpMRI-GTV (right) for the biochemical relapse (BR) and biochemical control (BC) groups

In addition, Fig. 3 illustrates the results of the comparison of near minimum (D98%) EQD2 for prostate gland and mpMRI-GTV. No significant differences between the two groups and for both target types were shown for prostate gland *p* = 0.0926 and for mpMRI-GTV *p* = 0.1895.

Finally, for completion purposes, the comparison of the dosimetric parameter values based on physical dose between the two groups (BR, BC) for the two targets (prostate gland, mpMRI-GTV) was performed. The results are available in the Additional file 1 (Sections 2 and 3).

An extended analysis considering a fixed *α*/*β* = 1.6 Gy at 5-year follow-up time and Gleason score (Additional file 1) confirmed the failure in prediction of response of any dosimetric index for mpMRI-GTV and prostate gland (see Additional file 1: Section 4).

### Linear-quadratic Poisson TCP model fitting

**Fig. 4:**
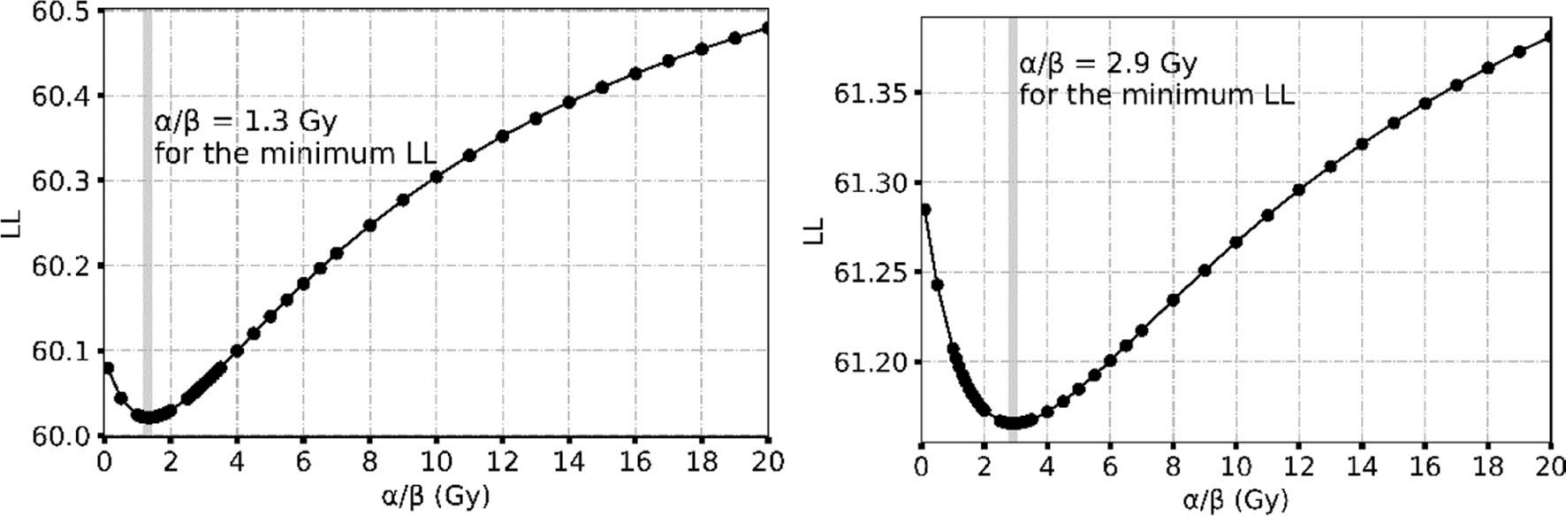
Plot of optimized *LL* (Eq. 10) against *α*/*β* when prostate gland (left) and mpMRI-GTV (right) are considered as the target causing the observed response. The *LL* level for the estimation of 95% CI of *α*/*β* is 63.86 for the prostate gland and 65.01 for the mpMRI-GTV

Due to the fact that there was no adequate variability in the fractionation scheme (dose per fraction in the range of 1.7 to 2.2 Gy) of the clinical data considered, it was not possible to define the 95% CI of *α*/*β* when considered as a free variable to be fitted. To analyse this effect, we investigated the behaviour of *LL* estimator values for *α*/*β* in the region of 0.1 Gy to 20.0 Gy. The results are presented in Fig. 4. The minimum *LL* values are observed for an *α*/*β* = 1.3 Gy when the prostate gland is considered as a target and *α*/*β* = 2.9 Gy when mpMRI-GTV is considered as the target defining the biological response. For a fine estimation of the minimum optimized *LL* value an α/β step of 0.1 Gy was considered. The fitness of the TCP model considering the prostate gland as underlying target is better than when mpMRI-GTV is considered as target: lower *LL* value is demonstrated for prostate gland than the mpMRI-GTV (Table 1): 60.02 versus 61.17. Evaluating the goodness-of-fit of the linear-quadratic Poisson TCP model using the Hosmer–Lemeshow (HL) test showed no significant difference between the observed and the predicted outcomes for both target types where for prostate gland the *HL* test showed *p* = 0.66 (*X*^2^ = 5.92) and for mpMRI-GTV a *p* = 0.54 (*X*^2^ = 7.00).

**Table 1:** Fitted clinical data to linear-quadratic Poisson TCP model for the two different target types for intermediate and high-risk prostate cancer patients, for the best estimated *α*/*β* values (Fig. 5)

Target type	*D*_50_ (Gy) [95% CI]	*γ* [95% CI]	*α*/*β* (Gy)	*LL*	*HL test*
Prostate gland	66.8 [64.6, 69.0]	3.81 [2.58, 5.20]	1.3 [0, ∞]	60.02	*p* = 0.66 (*X*^2^ = 5.92)
mpMRI-GTV	68.1 [66.1, 70.0]	4.45 [2.99, 6.12]	2.9 [0, ∞]	61.17	*p* = 0.54 (*X*^2^ = 7.00)

In the following analysis we assume for the two different target types, prostate gland and mpMRI-GTV, the above mentioned individual *α*/*β* values. For this case, the results of *LL*-based model fitting for the two target types are summarized in Table 1. For the TCP model based on prostate gland slightly lower *D*_50_ value and lower *γ* value than for mpMRI-GTV were estimated.

**Fig. 5:**
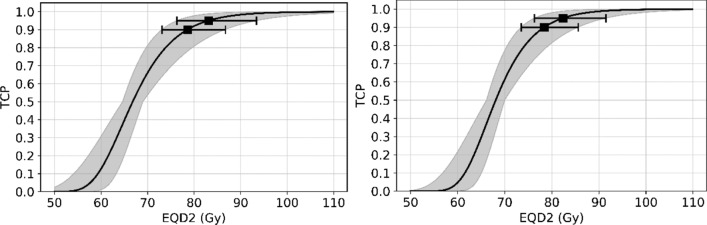
Response curves of linear-quadratic Poisson model for prostate gland with *α*/*β* = 1.3 Gy (left) and for mpMRI-GTV with *α*/*β* = 2.9 Gy (right). Grey area represents the 95% CI. The predicted *EQD2* values and their 95% CI for 90% and 95% TCP levels are also shown

The TCP predictions for the linear-quadratic Poisson TCP model for the two different target types in conjunction to EQD2 for homogeneous dose distribution are shown in Fig. 5.

The model predictions for TCP 90% and 95% biochemical response are for EQD2 values of homogeneous dose delivery of 78.6 Gy with 95% CI [73.1 Gy, 86.7 Gy] and 83.1 Gy with 95% CI [76.3 Gy, 93.4 Gy], respectively, when the prostate gland is considered as the responsible target. These values are slightly different when the mpMRI-GTV is considered as the target: 78.4 Gy with 95% CI [73.5 Gy, 85.6 Gy] and 82.4 Gy with 95% CI [76.3 Gy, 91.5 Gy], respectively.

**Fig. 6:**
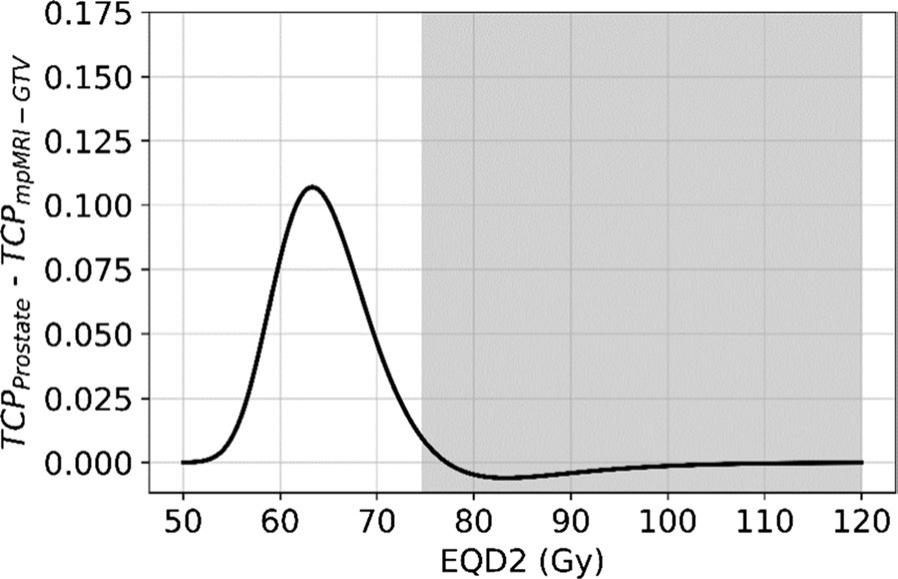
Absolute difference in predicted TCP values for the linear-quadratic Poisson model fitted for prostate gland (TCP_prostate_) and for mpMRI-GTV (TCP_mpMRI-GTV_). For *EQD2* above 74.6 Gy the absolute differences in TCP stay below 1%

The absolute difference of the TCP predictions of both linear-quadratic Poisson fitted models is shown in Fig. 6. For homogeneous dose delivery with *EQD2* values above 74.6 Gy (TCP ~ 0.81) both model predictions agree within 1%. The maximum TCP deviation (0.11) among the two models is observed for *EQD2* of 63.3 Gy.

**Fig. 7:**
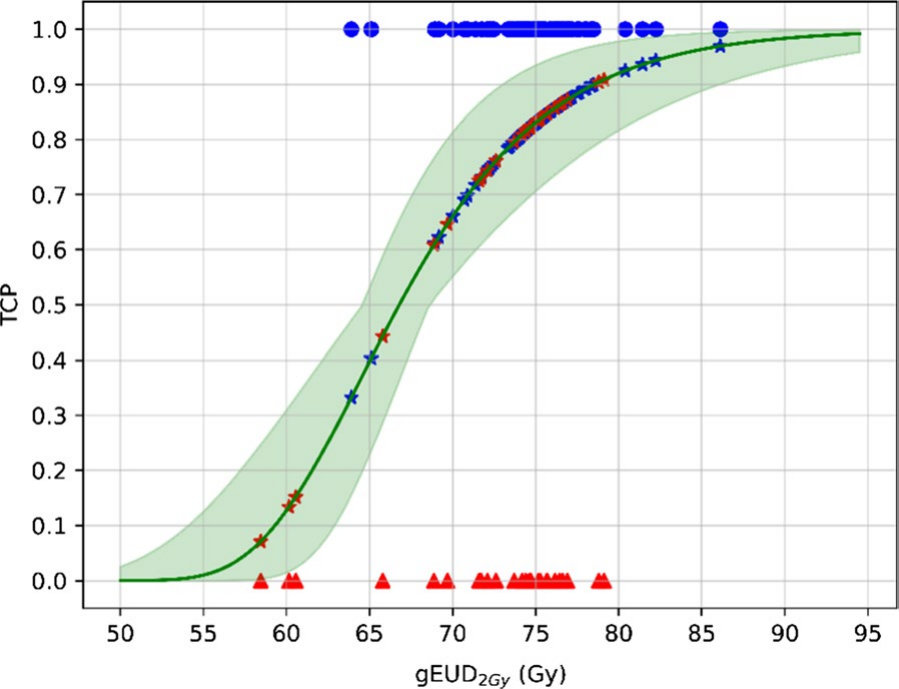
The red triangles represent BR patients, while the blue dots represent BC patients. For each patient the TCP has been calculated based on the dose distribution (DVH) to prostate gland. The model parameters were α volume effect = −10, *D*_50_ = 66.8 Gy, *γ* = 3.8 and *α*/*β* = 1.3 Gy. Shaded green area represents the 95% CI

In Fig. 7 we are showing the individual TCP values for the two response groups (BR, BC) based on the prostate gland planned dose distribution.

Finally, in addition to the previous analysis based on a mixed follow-up period, the clinical data has been re-fitted for a 5-year follow-up period after the radiation treatment. Our results for the prostate gland were *D*_50_ = 64.6 Gy [61.6 Gy, 67.4 Gy], *γ* = 3.1 [2.0, 4.4], *α*/*β* = 2.2 Gy (95% CI was undefined) (*LL*_*min*_ = 48.08). For the mpMRI-GTV, the optimizer was unable to deliver any reasonable results for the expected clinical *D*_50_ and *α*/*β*. The results for the mpMRI-GTV were *D*_50_ = 50.1 Gy [44.6 Gy, 56.0 Gy], *γ* = 0.8 [0.5, 1.2], *α*/*β* = 0.0 Gy (95% CI was undefined) (*LL*_*min*_ = 49.50).

Considering the most recent literature regarding *α*/*β* estimation based on large clinical cohorts by Vogelius and Bentzen [30], an *α*/*β* value of 1.6 Gy was found when no proliferation was considered and without any differentiation according to clinical stage or Gleason score. We repeated the TCP fitting using this *α*/*β* = 1.6 Gy and the results are presented in details in Additional file 1: Section 6. Regardless of any differentiation according to Gleason score or biochemical response at fixed 5-year follow-up time the *LL* estimator was always better for the prostate gland than for the mpMRI-GTV, confirming the results shown above for individually fitted *α*/*β* and mixed follow-up time.

Using this fixed *α*/*β* value for both targets and independently of follow-up time, the steepness of the TCP curve for mpMRI-GTV becomes lower than that for prostate gland with the only exception for Gleason score less than 8 and mixed follow-up time. This is also the case for the *D*_50_ at 5-year follow-up time.

Finally, we investigated the dependence of the estimated EQD2 values for 90% and 95% TCP on assumed *α*/*β* value and follow-up time. Detailed results are presented in Additional file 1: Section 5.2. Independently of the time follow-up Gleason score, the estimated EQD2 values for both target volumes are similar and close to the values previously listed for individually α/β and mixed follow-up time. The only exception is for fixed *α*/*β* = 1.6 Gy at 5-year follow-up and any Gleason score where the EQD2 value for 90% and 95% TCP for mpMRI-GTV are significantly higher than those for prostate gland and for any other investigated sub-group.

### Multivariate logistic TCP model fitting

For the multivariate logistic model, the full model (Eq. 9) was used. As stated previously the *gEUD*_*2Gy*_ for prostate gland was calculated with *α*/*β* = 1.3 Gy, and for mpMRI-GTV with *α*/*β* = 2.9 Gy. The best fitted nested model, after the stepwise analysis in both directions, showed only the *gEUD*_*2Gy*_ in prostate gland (*p* = 0.002) and the Gleason score (*p* = 0.003) to be significant, whereas the volume of mpMRI-GTV (*p* = 0.042) were marginally significant.

The goodness-of-fit as described by the *HL* test showed no significant difference between the observed and the predicted outcomes for the multivariate logistic model with *p* = 0.64 (*X*^2^ = 6.06).

When using a fixed α/β value of 1.6 Gy for both targets, the model fitting (HL-test: *p* = 0.7960, *X*^2^ = 4.63) shows the same results for a high significance for Gleason score (*p* = 0.0025) and *gEUD*_*2Gy*_ in prostate gland (*p* = 0.0021) and marginal significance for the volume of mpMRI-GTV (*p* = 0.0425) (Additional file 1: Section 6).

When a fixed *α*/*β* value of 1.6 Gy for both targets with clinical response data at 5-year follow-up are analysed with the multivariate logistic regression model (HL-test: *p* = 0.8424, *X*^2^ = 4.16), the significance of Gleason score (*p* = 0.039) is confirmed. Conversely, the gEUD2Gy for prostate gland (*p* = 0.0633) and the volume of mpMRI-GTV (*p* = 0.0775) has been shown to be marginally insignificant (Additional file 1: Section 6).

## Discussion

The analysis of minimum physical dose, minimum *EQD2* and *gEUD*_*2Gy*_ demonstrated significant lower values to the BR than to BC response group only for prostate gland. This is in alignment with the results of the multivariate logistic TCP model, demonstrating a significant predictive value only for the dose distribution in prostate gland expressed as *gEUD*_*2Gy*_. No dosimetric index could be identified to have significant predictive value when considering a fixed *α*/*β* = 1.6 Gy at 5-year follow-up time and differentiation according to Gleason score.

The analysis of *LL* fitness of the linear-quadratic Poisson TCP model in dependence on the assumed *α*/*β* value indicated two different *α*/*β* values as appropriate for the investigated target volume types: A very low *α*/*β* value of 1.3 Gy for prostate gland and a low *α*/*β* value of 2.9 Gy for mpMRI-GTV. These results are consistent with published estimations of *α*/*β* value for prostate cancer [1, 30–33]. However, our results indicated lower fractionation sensitivity for the mpMRI-GTV when this is considered to define the clinical response when compared to prostate gland.

Levegrün et al. [34] fitted TCP models with the maximum likelihood method to biopsy outcome from 103 prostate cancer patients with a minimum follow-up of 30 months, after 3D-CRT, using an *α*/*β* = 10.0 Gy and alternatively an *α*/*β* = 1.5 Gy. For their model fitting process individual DVHs for the planning target volume (PTV) recalculated for EQD2 have been used. Their results for *α*/*β* = 1.5 Gy were worse in terms of maximum likelihood values compared to the fitting results for *α*/*β* = 10.0 Gy for the low-, intermediate- and high-risk sub-groups. The estimated TCP model parameter values for *D*_50_ and *γ*_50_ for *α*/*β* = 10.0 Gy were 65.0 Gy and 2.9 (*γ* = 3.2) in the low-risk group, 67.8 Gy with 68% CI [64.7 Gy, 69.6 Gy] and 3.6 (*γ* = 4.0) in the intermediate-risk group and finally 75.7 Gy and 3.3 (*γ* = 3.7) in the high-risk group, respectively.

In contrast, we showed that for our cohort the linear-quadratic Poisson TCP model fits better for low *α*/*β* values (Fig. 5). The estimated *D*_50_ value of 66.8 Gy and 95% CI [64.6 Gy, 69.0 Gy] for our mixed intermediate- and high-risk cohort is lower than the corresponding values for those two risk groups in Levegrün’s publication. Our *γ* value of 3.8 with 95% CI [2.6, 5.2] lies in between the reported values for the two risk groups demonstrating similar steep TCP curves.

The differences observed in *D*_50_ values can be probably explained by the fact that Levegrün et al. used the PTV as responsible target which significantly overestimates the prostate gland due to the implemented setup margin of 10 mm [34, 35] and the higher assumed *α*/*β* value of 10.0 Gy compared to our estimation of 1.3 Gy for the prostate gland.

Fowler [36] published in 2005 a linear-quadratic logit model for 5-year biochemical control for intermediate risk prostate cancer patients, based on the prescription doses for prostate as given in the considered clinical data. Fowler estimated *D*_50_ = 65.6 Gy and *γ*_50_ = 2.1 (*γ* = 2.4) for *α*/*β* = 1.5 Gy with 95% CI [1.3 Gy-1.8 Gy]. Both values reported by Fowler, *D*_50_ and steepness of the dose response curve as described by *γ*, are lower compared to our results for prostate gland (*D*_50_ = 66.8 Gy, 95% CI [64.6 Gy, 69.0 Gy] and *γ* = 3.8, 95% CI [2.6, 5.2], Table 1). This is also the case when Fowler’s results are compared to the results by Levegrün et al. [34] for the intermediate-risk group. It must be pointed out that Fowler considered the prescription dose for his analysis whereas in the current study and in the paper by Levegrün et al. the individual planned DVHs for the target volumes have been utilized.

For our patient cohort the resulted range of minimum and maximum (variation) for the minimum physical dose, *EUD*, minimum *EQD2* and *gEUD*_*2Gy*_ for prostate gland were 28.0 Gy, 14.4 Gy, 43.0 Gy, 27.7 Gy accordingly. The observed variation in minimum physical dose of 28.0 Gy is much higher (factor of 2.3) than the range of planned prescription dose of 12.0 Gy (66.0 Gy to 78.0 Gy). Since the minimum dose in the target dominates the TCP, it is obvious that using the prescription dose for TCP modelling is a problematic simplification and biases the TCP model fitting expecting an underestimation of the steepness of the response curve.

The clinical results observed in our study can be better described by the linear-quadratic Poisson TCP model fitted to the dose distributions in the prostate gland than in mpMRI-GTV (*LL* = 60.02 for prostate gland versus *LL* = 61.17 for mpMRI-GTV).

This observation together with the failure to demonstrate significant differences in dosimetric parameters among BR and BC response groups when mpMRI-GTV is assumed as the responsible target could be explained by limitations of mpMRI to identify the true cancer volume in the prostate. As discussed by Zamboglou et al. [5], a better predictor of the biochemical response is the union region defined by mpMRI and PSMA PET. This is also supported by intra-individual comparison studies between mpMRI, PSMA PET and histopathology Refs. [37–40]. All these studies concluded that PSMA-PET provides superior detection of intraprostatic tumour lesions with better sensitivity than mpMRI. Thus, PSMA-PET/CT can be used to enhance mpMRI to provide improved detection and even characterization [41] of lesions.

Other probable explanations for these observations could be the limited number of 129 patients in our cohort, the low number of the observed failures (20%) and the relatively homogeneous dose per fraction in the range of 1.7 to 2.7 Gy. Another possible limitation in our analysis is the maximum six (6) month time of the mpMRI prior to radiation treatment, although prostate cancer is characterised by a slowly growing tumours with large doubling times. Furthermore, uncertainties in the delivered dose due to limitations of IGRT and intra-fractional movement which may have an influence on the presented results, have not been considered. Potential influence of factors such as ADT, radiation treatment technique, PSA serum level, time of mpMRI and clinical stage have been shown to be statistically insignificant (Cox regression analysis in Additional file 1: Section 1) regarding the described results.

Our findings regarding the failure to prove a benefit of using mpMRI-GTV in favour of prostate gland for predicting response are also confirmed when considering a fixed α/β = 1.6 Gy, a fixed follow-up time for biochemical response of 5 years or Gleason score differentiation. Finally, it should be noted that the novelty of the present study, in identifying the potential role of mpMRI-GTV to determine the clinical outcome, is that the planned DVHs for each patient have been considered instead of the prescription doses alone. To the best of our knowledge, only Levegrün et al. [34] considered individual planned DVHs for TCP modelling.

## Conclusion

In our study we observed 129 prostate cancer patients, who were treated with image-guided intensity modulated radiotherapy with a median clinical follow-up of 81.4 months (range 42.0–149.0). We estimated the radiobiological parameters of the linear-quadratic Poisson TCP model for prostate cancer patients for two relevant target structures, prostate gland and mpMRI-GTV considering the individually planned DVHs for the two targets and not simply the prescription doses of the clinical protocol. The model fits better to the clinical BRFS results when the prostate gland and not the mpMRI-GTV is considered as the underlying target and indicates a very low *α*/*β* = 1.3 Gy and a relative steep dose response curve (*γ* = 3.8 with 95% CI [2.6, 5.2]). A probable explanation could be limitations in defining GTV using mpMRI. This is also supported by the results of comparison of the dosimetric parameter values in both target types regarding biochemical response and by the fitting results of the multivariate logistic model.

Complementary to the previous analysis based on a mixed follow-up period, the clinical data has been re-fitted for a 5-year follow-up period after the radiation treatment. Our results for the prostate gland were *D*_50_ = 64.6 Gy [61.6 Gy, 67.4 Gy], *γ* = 3.1 [2.0, 4.4], *α*/*β* = 2.2 Gy (95% CI was undefined). For the mpMRI-GTV the optimizer was unable to deliver any reasonable results for the clinical expected *D*_50_ and *α*/*β*. The results for the mpMRI-GTV were *D*_50_ = 50.1 Gy [44.6 Gy, 56.0 Gy], *γ* = 0.8 [0.5, 1.2], *α*/*β* = 0.0 Gy (95% CI was undefined). The failure to identify any benefit for considering mpMRI-GTV as the target responsible for the clinical response is confirmed when taking into consideration a fixed *α*/*β* = 1.6 Gy, a fixed follow-up time for biochemical response at 5 years or Gleason score differentiation.

## Supplementary information

**Additional file 1.** Extended analysis.

## Data Availability

The datasets used and/or analyses during the current study are available from the corresponding author on reasonable request.

## Abbreviations

{*D*}: Dose distribution
3D: Three-dimensional
3D-CRT: Three-dimensional conformal radiation therapy
95% CI: 95% Confidence interval
ADT: Androgen deprivation therapy
BC: Biochemical control group
BFRS: Biochemical relapse free survival according to the Phoenix definition
BR: Biochemical relapse group
CT: Computed tomography
DDVH: Differential dose volume histograms
DIL: Dominant lesion
EBRT: External beam radiation therapy
EQD2: Equi-effective dose at 2 Gy per fraction
*gEUD*: Generalized equivalent uniform dose
GTV: Gross tumour volume
HL test: Hosmer–Lemeshow goodness-of-fit test
IGRT: Image-guided radiotherapy
IMRT: Intensity-modulated radiation therapy
*LL*: Log likelihood
LQ: Linear quadratic model
mpMRI: Multi-parametric MRI
MRI: Magnetic resonance imaging
*p*: *p* value for statistical hypothesis testing
PCa: Prostate cancer
PET: Positron emission tomography
PSA: Prostate-specific antigen (test)
PSMA: Prostate-specific membrane antigen
PTV: Planning target volume
RT: Radiotherapy
sd/SD: Standard deviation
TCP: Tumour control probability
VOI: Volume of interest
*α*/*β*: The ratio of ‘‘intrinsic radiosensitivity’’ to ‘‘repair capability’’ of a specified tissue
