# Supplementary material for: Prostate cancer tumour control probability modelling for external beam radiotherapy based on multi-parametric MRI-GTV definition
Source: Radiat Oncol. 2020 Oct 20;15:242. doi: 10.1186/s13014-020-01683-4 (PMC7574270; doi:10.1186/s13014-020-01683-4)

Supplementary material

# Analysis of the impact of the time of the mpMRI acquisition prior to the treatment and other factors

Regarding the time between MRI and begin of the treatment, we are presenting the histogram of MRI acquisition in days:


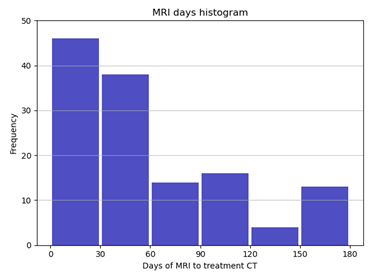


In addition, we run a cox regression analysis with a cut-off of 1 month between MRI and begin of EBRT, along with other covariates such as ADT, Radiation technique (IMRT, 3D-CRT), PSAGroups (1: <10ng/ml, 2: 10-20 ng/ml, 3: >20 ng/ml), Risikogruppe (0 for low, 1 for intermediate and 2 for high risk), GleasonScore (1 for <=7, 2 for 7 and 3 for => 8). The results of Cox regression indicated no higher risk for the patients who had MRI before 1 month to the ones who had MRI after 1 month, as well as for each covariate.


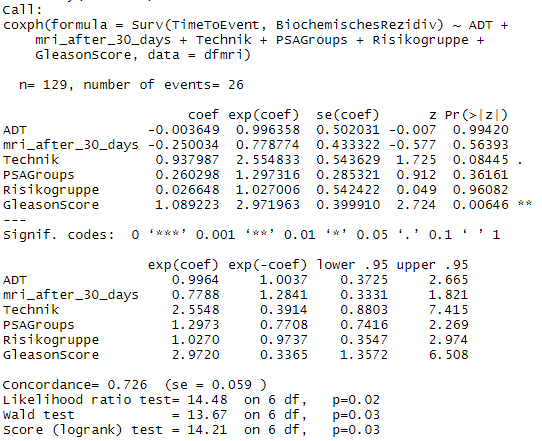


# Comparison of dose values in prostate gland and GTV

We compared the minimum physical dose for mpMRI-GTV and prostate gland for the biochemical control (BC), defined by BRFS, and biochemical relapse (BR) groups. For the BC group the median of the minimum dose in the prostate gland was 72.3 Gy (range 49.7 Gy – 77.7 Gy) whereas in the mpMRI-GTV it was 73.5 Gy (range 63.4 Gy – 78.4 Gy). For the BR group the median of the minimum dose was 71.3 Gy (range 54.2 Gy – 76.1 Gy) and 72.7 Gy (range 53.7 Gy – 76.0 Gy) for the prostate gland and mpMRI-GTV respectively.

As illustrated in Fig. 1, the minimum physical dose between the two groups is significantly different for prostate gland (*p* = 0.0345) but not for mpMRI-GTV (*p* = 0.0728).

| 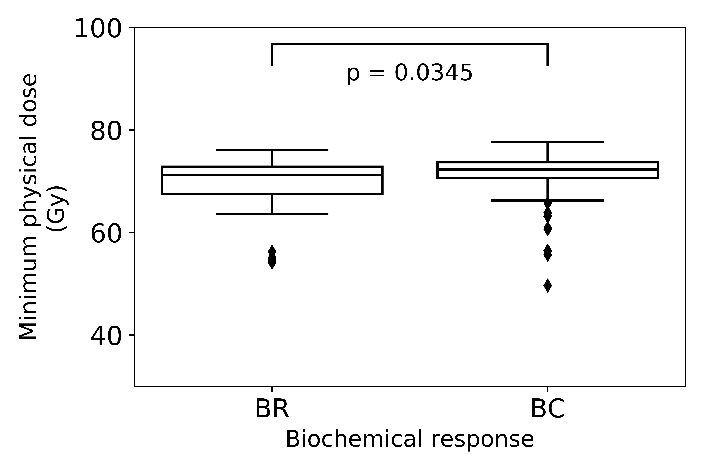 | 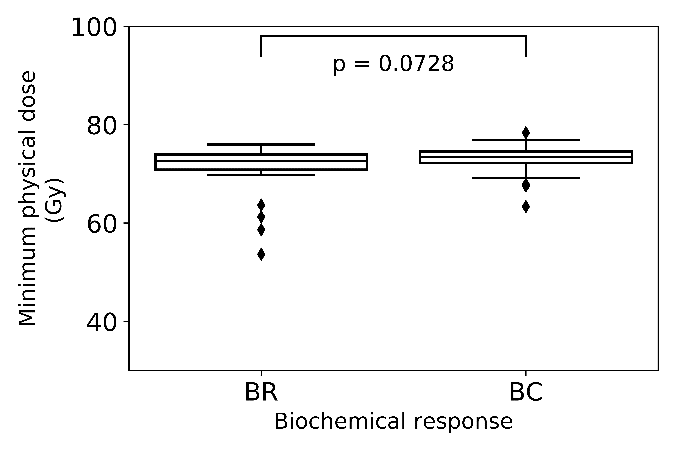 |
| --- | --- |
| **Figure 1**: Boxplots for the minimum physical dose for prostate gland (left, *p* = 0.0345) and mpMRI-GTV (right, *p* = 0.0728) for the biochemical control (BC) and biochemical relapse (BR) groups. | |

Regarding EUD and BC group the median value for the prostate gland was 75.5 Gy (range 68.3 Gy – 79.8 Gy) whereas for the mpMRI-GTV it was 75.3 Gy (range 69.8 Gy – 79.7 Gy). For the BR group the median EUD was 74.5 Gy (range 65.5 Gy – 77.2 Gy) and 74.5 Gy (range 65.6 Gy – 77.0 Gy) for the prostate gland and mpMRI-GTV respectively.

Figure 2 illustrates the results of the comparison of equivalent uniform dose for prostate gland and mpMRI-GTV. No significant differences between the two groups and for both target types were showed: for prostate gland *p* = 0.0859 and for mpMRI-GTV *p* = 0.1597.

| 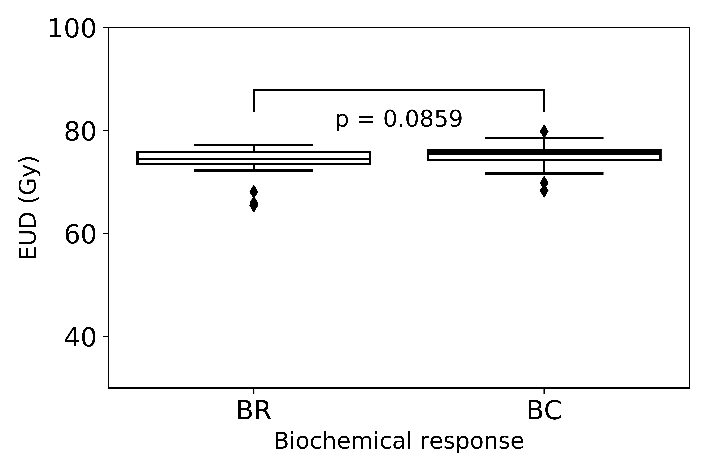 | 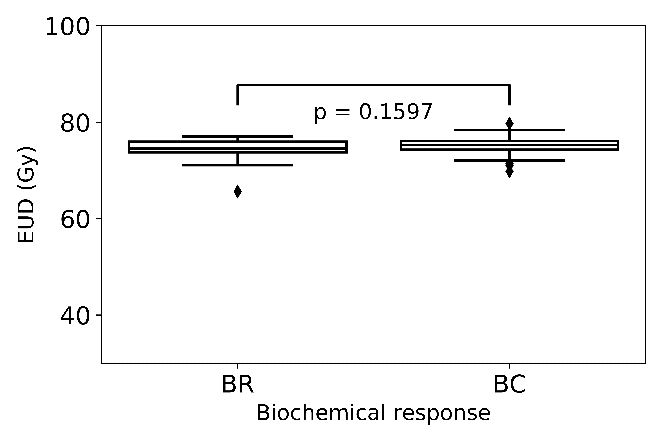 |
| --- | --- |
| **Figure 2**: Boxplots for the EUD for prostate gland (left) and mpMRI-GTV (right) for the biochemical relapse (BR) and biochemical control (BC) groups. | |

# Dose-Volume parameters for prostate gland and mpMRi-GTV

**Table 1**: Dose-Volume parameters for prostate gland and mpMRI-GTV for biochemical relapse (BR) group and biochemical control (BC) group. The Wilcoxon test for comparing the two groups is used with significance level α=0.05. (*) indicates a marginal significant difference, (**) indicates a strong significant difference

|  | **mpMRI-GTV** | | | **Prostate gland** | | |
| --- | --- | --- | --- | --- | --- | --- |
|  | Mean (SD) | | | Mean (SD) | | |
|  | BC | BR | Wilcoxon  p-value | BC | BR | Wilcoxon  p-value |
| Dmin [Gy] | 73.12 (2.08) | 70.89 (5.35) | 0.07 | **71.2(4.19)** | **68.55(6.34)** | **0.03(**)** |
| Dmax [Gy] | 76.83 (1.71) | 75.97 (2.76) | 0.22 | 77.91(1.84) | 77.03(2.52) | 0.12 |
| Dmean [Gy] | 75.16 (1.50) | 74.26 (2.78) | 0.17 | 75.21(1.53) | 74.19(2.62) | 0.10 |
| D98% [Gy] | 73.85 (1.81) | 72.18 (4.39) | 0.20 | 77.33(2.31) | 71.21(4.96) | 0.10 |
| D50% [Gy] | 75.18 (1.49) | 74.34 (2.78) | 0.23 | 75.25(1.49) | 74.30(2.50) | 0.12 |
| D2% [Gy] | 76.35 (1.62) | 75.53 (2.68) | 0.28 | 76.79(1.59) | 76.04(2.25) | 0.13 |
| D70% [Gy] | 74.83 (1.49) | 73.98 (2.82) | 0.22 | 74.80(1.55) | 73.67(2.94) | 0.13 |
| D80% [Gy] | 74.63 (1.49) | 73.75 (2.85) | 0.24 | 74.52(1.60) | 73.31(3.22) | 0.16 |
| D65% [Gy] | 74.92 (1.49) | 74.08 (2.80) | 0.24 | 74.92(1.53) | 73.82(2.83) | 0.12 |
| D60% [Gy] | 75.01 (1.49) | 74.17 (2.79) | 0.23 | 75.03(1.52) | 73.98(2.72) | 0.11 |
| D35% [Gy] | 75.33 (1.50) | 74.59 (2.76) | 0.20 | 75.57(1.48) | 74.75(2.31) | 0.14 |
| D30% [Gy] | 75.53 (1.51) | 74.67 (2.74) | 0.21 | 75.69(1.48) | 74.89(2.28) | 0.14 |
| D15% [Gy] | 75.83 (1.56) | 74.99 (2.68) | 0.22 | 76.08(1.50) | 75.33(2.23) | 0.12 |
| D25% [Gy] | 75.62 (1.53) | 74.77 (2.68) | 0.22 | 75.80(1.49) | 75.03(2.26) | 0.13 |
| D3% [Gy] | 76.27 (1.61) | 75.45 (2.68) | 0.28 | 76.67(1.57) | 75.95(2.25) | 0.13 |
| D1cc [Gy] | 62.40 (28.38) | 63.33 (26.89) | 0.89 | 76.78(1.58) | 76.04(2.24) | 0.12 |
| D0.1cc [Gy] | 76.20 (1.63) | 75.43 (2.76) | 0.32 | 77.27(1.67) | 76.49(2.31) | 0.12 |

# Dose-Volume parameters for prostate gland and mpMRI-GTV for biochemical response at 5 years

## Gleason score: any

|  | **mpMRI-GTV** | | | **Prostate gland** | | |
| --- | --- | --- | --- | --- | --- | --- |
|  | Mean (SD) | | | Mean (SD) | | |
|  | BC | BR | Wilcoxon  p-value | BC | BR | Wilcoxon  p-value |
| Dmin [Gy] | 72.60 (3.16) | 71.75 (3.97) | 0.48 | 70.89 (4.29) | 69.19 (5.37) | 0.23 |
| Dmax [Gy] | 76.68 (1.95) | 76.23 (2.71) | 0.59 | 77.79 (1.86) | 76.99 (2.86) | 0.34 |
| Dmean [Gy] | 74.98 (1.83) | 74.52 (2.46) | 0.52 | 75.08 (1.65) | 74.23 (2.71) | 0.28 |
| D98% [Gy] | 73.50 (2.61) | 72.75 (3.32) | 0.55 | 73.02 (2.74) | 71.39 (4.72) | 0.27 |
| D50% [Gy] | 75.00 (1.83) | 74.61 (2.45) | 0.61 | 75.13 (1.61) | 74.35 (2.59) | 0.35 |
| D2% [Gy] | 76.20 (1.86) | 75.73 (2.59) | 0.59 | 76.70 (1.63) | 76.00 (2.54) | 0.36 |
| D70% [Gy] | 74.64 (1.85) | 74.23 (2.43) | 0.59 | 74.67 (1.68) | 73.69 (2.99) | 0.32 |
| D80% [Gy] | 74.44 (1.86) | 73.98 (2.42) | 0.61 | 74.38 (1.76) | 73.33 (3.22) | 0.35 |
| D65% [Gy] | 74.74 (1.84) | 74.34 (2.43) | 0.63 | 74.79 (1.65) | 73.85 (2.89) | 0.30 |
| D60% [Gy] | 74.82 (1.83) | 74.44 (2.44) | 0.61 | 74.91 (1.64) | 74.02 (2.79) | 0.30 |
| D35% [Gy] | 75.27 (1.82) | 74.85 (2.48) | 0.56 | 75.45 (1.59) | 74.83 (2.44) | 0.41 |
| D30% [Gy] | 75.36 (1.82) | 74.92 (2.49) | 0.54 | 75.57 (1.59) | 74.97 (2.43) | 0.43 |
| D15% [Gy] | 75.67 (1.83) | 75.20 (2.52) | 0.52 | 75.97 (1.58) | 75.38 (2.46) | 0.42 |
| D25% [Gy] | 75.45 (1.82) | 75.00 (2.50) | 0.55 | 75.69 (1.58) | 75.10 (2.43) | 0.44 |
| D3% [Gy] | 76.12 (1.85) | 75.65 (2.57) | 0.57 | 76.58 (1.62) | 75.91 (2.53) | 0.35 |
| D1cc [Gy] | 61.86 (28.67) | 63.23 (27.17) | 0.97 | 76.67 (1.63) | 76.00 (2.52) | 0.35 |
| D0.1cc [Gy] | 76.04 (1.89) | 75.66 (2.61) | 0.66 | 77.18 (1.71) | 76.43 (2.61) | 0.32 |
| D98% EQD2 (*) | 72.71 (4.3) | 71.23 (4.86) | 0.34 | 71.97 (4.38) | 69.27 (6.78) | 0.21 |
| gEUD2Gy (*,**) | 74.79 (3.41) | 73.71 (3.95) | 0.27 | 74.86 (3.17) | 72.80 (5.10) | 0.12 |

*for fixed α/β=1.6Gy, ** for a=-10

## Gleason score: <8

|  | **mpMRI-GTV** | | | **Prostate gland** | | |
| --- | --- | --- | --- | --- | --- | --- |
|  | Mean (SD) | | | Mean (SD) | | |
|  | BC | BR | Wilcoxon  p-value | BC | BR | Wilcoxon  p-value |
| Dmin [Gy] | 72.78 (2.50) | 70.53 (5.00) | 0.23 | 71.09 (3.72) | 68.75 (5.49) | 0.19 |
| Dmax [Gy] | 76.83 (1.93) | 75.15 (2.97) | 0.06 | 77.97 (1.79) | 76.12 (3.28) | 0.08 |
| Dmean [Gy] | 75.06 (1.81) | 73.71 (2.99) | 0.13 | 75.19 (1.56) | 73.47 (3.32) | 0.15 |
| D98% [Gy] | 73.68 (2.14) | 71.70 (4.16) | 0.22 | 73.25 (2.18) | 70.71 (5.74) | 0.25 |
| D50% [Gy] | 75.07 (1.82) | 73.82 (2.99) | 0.16 | 75.23 (1.55) | 73.65 (3.13) | 0.16 |
| D2% [Gy] | 76.32 (1.84) | 74.72 (2.95) | 0.06 | 76.83 (1.57) | 75.27 (3.06) | 0.10 |
| D70% [Gy] | 74.72 (1.84) | 73.51 (3.00) | 0.21 | 74.75 (1.62) | 72.86 (3.67) | 0.16 |
| D80% [Gy] | 74.51 (1.86) | 73.27 (3.00) | 0.21 | 74.46 (1.70) | 72.45 (4.00) | 0.18 |
| D65% [Gy] | 74.81 (1.83) | 73.60 (3.00) | 0.21 | 74.88 (1.59) | 73.05 (3.53) | 0.16 |
| D60% [Gy] | 74.90 (1.82) | 73.69 (2.99) | 0.18 | 75.00 (1.58) | 73.24 (3.39) | 0.15 |
| D35% [Gy] | 75.35 (1.81) | 74.01 (2.99) | 0.12 | 75.56 (1.53) | 74.21 (2.95) | 0.20 |
| D30% [Gy] | 75.44 (1.81) | 74.08 (2.99) | 0.11 | 75.68 (1.52) | 74.35 (2.95) | 0.20 |
| D15% [Gy] | 75.76 (1.82) | 74.31 (2.98) | 0.10 | 76.10 (1.51) | 74.73 (2.99) | 0.19 |
| D25% [Gy] | 75.54 (1.81) | 74.15 (2.99) | 0.12 | 75.18 (1.52) | 74.48 (2.96) | 0.20 |
| D3% [Gy] | 76.23 (1.83) | 74.66 (2.95) | 0.06 | 76.72 (1.55) | 75.20 (3.05) | 0.12 |
| D1cc [Gy] | 61.59 (28.96) | 66.79 (22.26) | 0.74 | 76.82 (1.56) | 75.29 (3.02) | 0.10 |
| D0.1cc [Gy] | 76.15 (1.88) | 74.70 (3.00) | 0.11 | 77.34 (1.65) | 75.64 (3.08) | 0.07 |
| D98% EQD2 (*) | 72.77 (3.50) | 69.96 (5.97) | 0.19 | 72.12 (3.49) | 68.60 (8.03) | 0.30 |
| gEUD2Gy (*,**) | 74.78 (3.07) | 72.71 (4.47) | 0.12 | 74.88 (2.78) | 71.82 (6.03) | 0.18 |

*for fixed α/β=1.6Gy, ** for a=-10

## Gleason score: ≥8

|  | **mpMRI-GTV** | | | **Prostate gland** | | |
| --- | --- | --- | --- | --- | --- | --- |
|  | Mean (SD) | | | Mean (SD) | | |
|  | BC | BR | Wilcoxon  p-value | BC | BR | Wilcoxon  p-value |
| Dmin [Gy] | 71.56 (5.54) | 73.11 (1.41) | 0.84 | 69.78 (6.59) | 69.68 (5.18) | 0.84 |
| Dmax [Gy] | 75.84 (1.84) | 77.44 (1.72) | 0.08 | 76.76 (1.88) | 77.95 (1.87) | 0.29 |
| Dmean [Gy] | 74.47 (1.85) | 75.43 (1.13) | 0.32 | 74.42 (1.98) | 75.08 (1.38) | 0.65 |
| D98% [Gy] | 72.45 (4.28) | 73.93 (1.21) | 0.56 | 71.67 (4.63) | 72.15 (3.07) | 1.00 |
| D50% [Gy] | 74.55 (1.82) | 75.48 (1.14) | 0.26 | 74.54 (1.84) | 75.13 (1.46) | 0.51 |
| D2% [Gy] | 75.51 (1.85) | 76.86 (1.44) | 0.14 | 75.88 (1.74) | 76.80 (1.42) | 0.32 |
| D70% [Gy] | 74.22 (1.84) | 75.04 (1.11) | 0.39 | 74.15 (1.91) | 74.61 (1.52) | 0.56 |
| D80% [Gy] | 74.04 (1.84) | 74.77 (1.10) | 0.36 | 73.89 (1.99) | 74.31 (1.53) | 0.60 |
| D65% [Gy] | 74.31 (1.84) | 75.16 (1.12) | 0.32 | 74.26 (1.89) | 74.74 (1.51) | 0.56 |
| D60% [Gy] | 74.39 (1.83) | 75.27 (1.13) | 0.29 | 74.36 (1.87) | 74.88 (1.49) | 0.56 |
| D35% [Gy] | 74.79 (1.81) | 75.78 (1.20) | 0.19 | 74.81 (1.81) | 75.52 (1.39) | 0.43 |
| D30% [Gy] | 74.88 (1.81) | 75.87 (1.22) | 0.23 | 74.90 (1.79) | 75.66 (1.37) | 0.43 |
| D15% [Gy] | 75.15 (1.83) | 76.19 (1.28) | 0.23 | 75.22 (1.75) | 76.11 (1.33) | 0.36 |
| D25% [Gy] | 74.96 (1.81) | 75.96 (1.24) | 0.26 | 74.99 (1.78) | 75.80 (1.36) | 0.43 |
| D3% [Gy] | 75.46 (1.85) | 76.75 (1.40) | 0.16 | 75.78 (1.73) | 76.70 (1.40) | 0.32 |
| D1cc [Gy] | 63.51 (26.81) | 59.28 (31.28) | 0.69 | 75.82 (1.73) | 76.79 (1.44) | 0.29 |
| D0.1cc [Gy] | 75.44 (1.88) | 76.72 (1.48) | 0.19 | 76.25 (1.79) | 77.30 (1.54) | 0.19 |
| D98% EQD2 (*) | 72.34 (7.41) | 72.64 (2.54) | 0.65 | 71.10 (7.67) | 70.02 (4.91) | 0.43 |
| gEUD2Gy (*,**) | 74.82 (4.87) | 74.83 (2.57) | 0.90 | 74.73 (4.80) | 73.89 (3.07) | 0.65 |

*for fixed α/β=1.6Gy, ** for a=-10

# TCP fitting results with fixed α/β=1.6Gy for mixed follow-up and 5 years

## Model fitting results

| Target type | *D_50_* (Gy)  [95%CI] | *γ*  [95%CI] | *α/β* (Gy) | *LL* | *Follow-up time* | *Patients*  *number* | *Events number* | *Gleason score* |
| --- | --- | --- | --- | --- | --- | --- | --- | --- |
| prostate gland | 67.13  [65.0, 69.2] | 4.0  [2.7, 5.4] | 1.6 | 60.02 | Mixed time | 129 | 26 | any |
| mpMRI-GTV | 67.10  [64.86, 69.20] | 3.8  [2.6, 5.3] | 1.6 | 61.18 | Mixed time | 129 | 26 | any |
| prostate gland | 65.41  [62.76, 67.8] | 4.4  [3.1, 6.1] | 1.6 | 36.03 | Mixed time | 103 | 14 | < 8 |
| mpMRI-GTV | 65.96  [63.46, 68.22] | 4.7  [3.2, 6.6] | 1.6 | 36.77 | Mixed time | 103 | 14 | < 8 |
| prostate gland | 63.9  [60.8, 67.0] | 2.9  [1.9, 4.1] | 1.6 | 48.08 | 5 years | 108 | 19 | any |
| mpMRI-GTV | 56.3  [51.6, 61.1] | 1.3  [0.9, 1.9] | 1.6 | 49.55 | 5 years | 108 | 19 | any |
| prostate gland | 62.96  [59.54 ,66.12] | 3.6  [2.4, 5.1] | 1.6 | 28.29 | 5 years | 86 | 10 | < 8 |
| mpMRI-GTV | 59.18  [54.78, 63.32] | 2.4  [1.6, 3.5] | 1.6 | 29.68 | 5 years | 86 | 10 | < 8 |

## EQD2 values for 90% and 95% TCP for prostate gland and mpMRI-GTV

| Target type | EQD2 for  TCP=90%  (min, max) | EQD2 for  TCP=95%  (min, max) | α/β (Gy) | Follow-up time | Gleason score |
| --- | --- | --- | --- | --- | --- |
| prostate gland | 78.6  (73.1, 86.7) | 83.1  (76.3, 93.4) | 1.3 | Mixed time | any |
| mpMRI-GTV | 78.4  (73.5, 85.6) | 82.4  (76.3, 91.5) | 2.9 | Mixed time | any |
| prostate gland | 78.4  (73.2, 86.2) | 82.7  (76.3, 92.6) | 1.6 | Mixed time | any |
| mpMRI-GTV | 77.3  (72.1, 84.5) | 81.2  (74.9, 90.4) | 1.6 | Mixed time | any |
| prostate gland | 75.5  (69.8, 82.3) | 79.3  (72.5, 87.9) | 1.6 | Mixed time | < 8 |
| mpMRI-GTV | 75.5  (70.0, 82.4) | 79.1  (72.5, 87.8) | 1.6 | Mixed time | < 8 |
| prostate gland | 78.5  (70.8, 89.9) | 84.1  (74.6, 98.6) | 1.6 | 5 years | any |
| mpMRI-GTV | 83.5  (69.3, 101.9) | 93.9  (76.1, 117.5) | 1.6 | 5 years | any |
| prostate gland | 74.7  (67.5, 84.1) | 79.2  (70.5, 91.0) | 1.6 | 5 years | < 8 |
| mpMRI-GTV | 75.4  (65.3, 88.6) | 81.6  (69.3, 98.3) | 1.6 | 5 years | < 8 |

# Results for logistic regression model including Gleason

## Mixed follow-up time for α/β=1.3Gy for prostate gland and α/β=2.9Gy for mpMRI-GTV


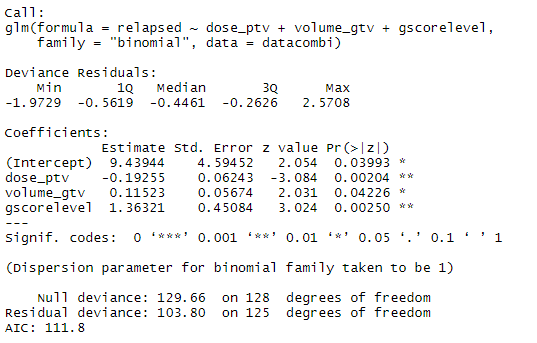


## Mixed follow-up time for α/β=1.6Gy

##
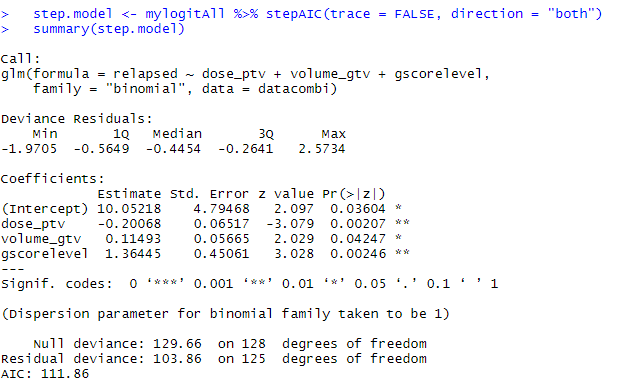


## At 5 Years for α/β=1.6Gy


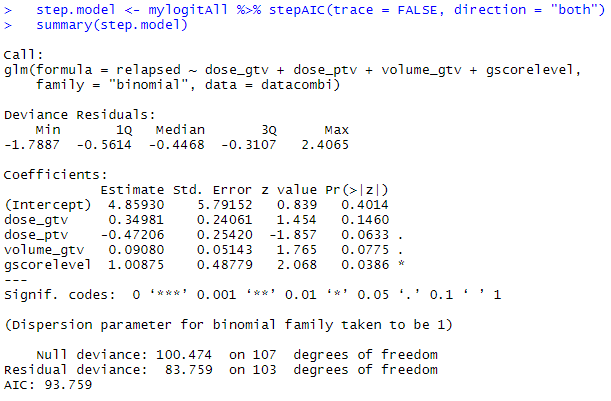

Supplement: Supplementary file 1 — Additional file 1. Extended analysis. [file 13014_2020_1683_MOESM1_ESM.docx]
